# Social Cognition in Anorexia Nervosa: Evidence of Preserved Theory of Mind and Impaired Emotional Functioning

**DOI:** 10.1371/journal.pone.0044414

**Published:** 2012-08-31

**Authors:** Mauro Adenzato, Patrizia Todisco, Rita B. Ardito

**Affiliations:** 1 Center for Cognitive Science, Department of Psychology, University of Turin, Turin, Italy; 2 Neuroscience Institute of Turin, Turin, Italy; 3 Center of Eating Disorders, Casa di Cura Villa Margherita, Arcugnano, Vicenza, Italy; University of Bologna, Italy

## Abstract

**Background:**

The findings of the few studies that have to date investigated the way in which individuals with Anorexia Nervosa (AN) navigate their social environment are somewhat contradictory. We undertook this study to shed new light on the social-cognitive profile of patients with AN, analysing Theory of Mind and emotional functioning. Starting from previous evidence on the role of the amygdala in the neurobiology of AN and in the social cognition, we hypothesise preserved Theory of Mind and impaired emotional functioning in patients with AN.

**Methodology:**

Thirty women diagnosed with AN and thirty-two women matched for education and age were involved in the study. Theory of Mind and emotional functioning were assessed with a set of validated experimental tasks. A measure of perceived social support was also used to test the correlations between this dimension and the social-cognitive profile of AN patients.

**Principal Findings:**

The performance of patients with AN is significantly worse than that of healthy controls on tasks assessing emotional functioning, whereas patients’ performance is comparable to that of healthy controls on the Theory of Mind task. Correlation analyses showed no relationship between scores on any of the social-cognition tasks and either age of onset or duration of illness. A correlation between social support and emotional functioning was found. This latter result seems to suggest a potential role of social support in the treatment and recovery of AN.

**Conclusions:**

The pattern of results followed the experimental hypothesis. They may be useful to help us better understand the social-cognitive profile of patients with AN and to contribute to the development of effective interventions based on the ways in which patients with AN actually perceive their social environment.

## Introduction

Anorexia nervosa (AN) is a severe mental disorder characterised by refusal to maintain body weight at or above a minimally normal level for age and height, intense fear of gaining weight or becoming fat, and disturbance in the way in which one’s body weight or shape is experienced [1]. Up to 80% of sufferers (predominantly females) engage in excessive exercise, in addition to self-starvation, to reduce their body weight [2]–[3]. The etiology of AN is not fully understood, but a crucial role is seemingly played by the complex interplay among biological, genetic, psychological, and socio-cultural factors [4]. AN has a prevalence of about 0.3% in young women [5] and has the highest mortality rate among psychiatric disorders [6]. Two forms of AN are described in the DSM-IV [1], the restrictive subtype (AN-R) and the binge-purge subtype (AN-BP). AN-R subtype refers to individuals who restrict food without regularly engaging in binge-eating or purging behavior (i.e. self-induced vomiting or the misuse of laxatives, diuretics, or enemas) while the AN-BP subtype refers to individuals who regularly engage in binge-eating or purging behavior. The distinction between these two subtypes has been questioned by authors that have performed longitudinal study indicating that the migration from a diagnostic category to another is very common between AN subtypes [7]–[8].

In sharp contrast to other psychiatric [9]–[13] and neuropsychological disorders [14]–[18], in which social-cognitive competences have already been investigated extensively and for a long time, only recently have researchers and clinicians started to investigate the domain of social cognition in patients with AN. Social cognition is the ability to construct mental representations of the relations that exist between oneself and others and to flexibly use these representations to function effectively in one’s social environment [19]–[20]. Social cognition is a sum of different processes and depends on the exchange of specific signals such as facial expression, body movement, and eye gaze [21]. Examples of abilities referable to the domain of social cognition are both our capacity to represent other people’s intentions and beliefs (i.e. Theory of Mind) [22]–[23], and the capacity to share the emotions and sensations of others (i.e. empathy) [24]–[25]. These distinct capacities display different ontogenetic trajectories reflecting the different developmental pathways of their underlying neural structures and represent the bases of any interpersonal relations [26]–[27].

The findings of the few studies that have to date investigated the way in which individuals with AN navigate their social environment are somewhat contradictory. With regard to Theory of Mind (ToM), Tchanturia et al. [28] assessed whether individuals with AN show a deficit in their ToM ability by administering to 20 female participants with AN two ToM tasks–a story-comprehension task previously employed to study children with autism [29] and a cartoon task previously employed to measure ToM in stroke patients [30]. Their findings show that the proportion of individuals who perform poorly on ToM tasks is higher among women with AN than among matched healthy controls, but also show that AN participants have impairments in both the ToM and control tasks. The authors thus suggest no evidence of any selective ToM impairment in AN sufferers. In a next study, Hambrook and Tchanturia [31] have explored Machiavellian competence in AN. Machiavellianism is the tendency to deceive and manipulate others for personal gain, and correlates with high manipulativeness, insincerity, and callousness [32]. This competence is closely connected to ToM ability, as it requires the comprehension of others’ thoughts and intentions in order to predict how they will behave and how one can manipulate them. To study Machiavellianism in people with AN, Hambrook and Tchanturia [31] used the Mach-IV, a self-report questionnaire composed of 20 statement items. Their results indicated no significant differences between people with AN and healthy controls. In contrast with these findings, more recently, Russell et al. [33] found ToM impairments in the Reading the Mind in the Eyes task (RME, see below for a detailed description of this task) in a group of 22 patients with AN. This last result was not replicated by Oldershaw et al. [34], who did not find impaired performance in the RME task in people with AN, but did find impairments in two different versions of this task (the voice and films containing a social scene versions). Interestingly, in two studies by Harrison and her colleagues [35]–[36] people with AN performed poorer than healthy controls on the RME task, while in a further work [37] these authors found only a statistical trend (*p* = .091) toward a significant difference between AN patients of the restrictive subtype and healthy controls and no difference for the binge-purge subtype.

Conflicting results were also found by Hambrook et al. [38]. They predicted that AN patients would display an empathising/systemising psychometric profile similar to that found in people with autism spectrum disorders. To this end, three standardised self-report measures were administered: the Autism-Spectrum Quotient [39], the Systemizing Quotient [40] and the Empathy Quotient [41]. The results revealed that women with AN and healthy controls did not differ in scores on either the Empathy Quotient or the Systemizing Quotient, but at the same time, that AN patients did report significantly higher total Autism-Spectrum Quotient scores than did healthy controls. According to Hambrook et al. [38], this latter result suggests that AN patients experience difficulties similar to those found within the autism spectrum disorders.

With regard to emotional functioning, Guttman and Laporte [42], using the Interpersonal Reactivity Index [43], an instrument for assessing four dimensions of empathy, did not find any difference between AN patients and healthy controls; however, empathy disorders were found in AN patients by Gillberg et al. [44] on the basis of structured clinical interviews. Interestingly, these two studies converge in ascribing to individuals with AN alexithymic disorders, the single rather well-established result in the AN literature concerning social cognition [45]–[47]. Furthermore, although different studies have found in people with AN difficulty recognising emotions from facial expression and vocal tone [48]–[50], recently Rozenstein et al. [51] have shown that patients with AN are as fast and as accurate in matching both facial identity and facial emotions as their unaffected sisters and unrelated healthy controls. In an attempt to systematize this literature, Zucker et al. [52] generated the hypothesis that individuals with AN fail to process facial regions as a result of elevated levels of anxiety around emotional experience that lead to prolonged avoidance of salient facial cues, promoting inaccurate interpretation. According to these authors, such chronic avoidance would lead to decreased experience in the processing of faces and increased dysfunction over time.

Faced with the discordant literature described above, we undertook this study to shed new light on the social-cognitive profile of patients with AN, analysing ToM and emotional functioning. To this end, we used a set of validated experimental tasks widely employed in the social-cognition literature: the Reading the Mind in the Eyes (RME) test, the Empathy Quotient (EQ), and the Twenty-Item Toronto Alexithymia Scale (TAS-20) (see below for a detailed description of these tasks). Furthermore, as a potential role of social support in the treatment and recovery of AN was proposed [53], we also used the Multidimensional Scale of Perceived Social Support (MSPSS) with the aim to explore this proposal and to check for possible correlations between the social-cognitive profile and the social support subjectively perceived by the patients with AN.

The experimental hypothesis of the present study is based on the neuroimaging and neuropsychological evidence that ToM and emotional functioning are associated with overlapping but distinct brain networks [54]–[56]. Common areas of activation are the prefrontal cortex, the superior temporal sulcus, and the temporo-parietal junctions. These areas form the basis for making inferences about mental states [57]–[59]. However, the appreciation of the emotional states requires the additional engagement of emotional networks, and particularly of the amygdala [55]–[56], [60]–[61]. In fact, while the amygdala is not involved in mentalising (i.e. ToM) *per se* [62] and is not necessary for ToM expression [63]–[65], its role in processing basic and social emotions, related both to the self and to others, is well known [27], [55], [65]–[67]. Interestingly, an increasing number of neuroimaging studies [68]–[77], adopting different experimental paradigms, have shown structural and functional alterations of the amygdala in people with AN and have highlighted the crucial role of this brain structure in the neurobiology of AN. Thus, starting from the neuroimaging evidence according to which a) the amygdala plays a pivotal role both in the neurobiology of AN and in all facets of emotional processing, and b) the amygdala is not necessary for ToM, in the present work we hypothesise that the performance of patients with AN will be significantly worse than that of healthy controls on tasks assessing emotional functioning (i.e. the EQ and the TAS-20), whereas AN patients’ performance will be comparable to that of healthy controls on the ToM task (i.e. the RME).

## Methods

### 1. Ethics Statement

The study was approved by the Spedali Civili Hospital of Brescia’s ethics committee and was conducted in accordance with the Declaration of Helsinki. Written informed consent was obtained from all participants, and written parental permission was requested and received for participants under 18.

### 2. Participants

Thirty women diagnosed with AN according to the DSM-IV [1] were recruited from the Regional Center of Eating Disorders at the Spedali Civili Hospital of Brescia, Italy. Sixteen participants fell into the restrictive subtype (AN-R), and 14 into the binge-purge subtype (AN-BP) at the time of the study. Consensus diagnoses were obtained from senior psychiatrists who assessed the patients in clinical interviews and by use of the Structured Clinical Interview for DSM-IV [78]. Exclusion criteria were comorbidities of the following types: mental retardation, cognitive disorders, psychosis, major depression, and personality disorders.

Thirty-two women were involved in the healthy control (HC) group. Exclusion criteria for the HC group were the presence or history of a neurological or psychiatric disorder, substance abuse, or dependence. Furthermore, a HC participant was included in the study only when she had both a Body Mass Index (BMI = Kg/m^2^) between 18.50 and 24.99 (i.e. within the normal range of weight according to the World Health Organization [79]), and a score below the cut-off of 30 on the Eating Attitudes Test [80], a standardised 40-item self-report scale measuring the risk of an eating disorders. All the participants were native Italian speakers.

### 3. Procedure

All participants were tested individually in a quiet room. Testing required one session lasting about 45–60 minutes. Tasks were administered to all participants in random order. BMI was calculated based on the weight and height of participants measured on the day of testing.

### 4. Materials

#### 4.1 Reading the mind in the eyes (RME) test

The RME is an advanced ToM test measuring an adult’s mentalising ability [81]. In the test, the experimenter presents a set of 36 photographs of the eye region of various human faces. Participants are required to choose among four words that are printed on the page that the picture appears on, using the criterion of which word best describes the mental state of the person depicted in the photograph. Participants have unlimited time to decide, and a glossary is provided. In order to pass this test, participants have to put themselves into the mind of another person and recognize his or her complex mental state, an ability that emerges around the time of adolescence. In the gender-recognition control task, participants are asked to judge the gender of the person in each of the 36 photographs. For both experimental (mental state attribution) and control (gender attribution) conditions, the maximum score is 36.

The RME test is administrable in adults at least 16 years old. For this reason, we excluded participants below this age from both the AN and HC groups.

#### 4.2 Empathy quotient (EQ)

The EQ is a validated self-report questionnaire for the assessment of the capacity to empathize with other, i.e. to recognize another’s affective state and to respond with an appropriate emotion to this [41]. The EQ contains 40 empathy items and 20 filler items included to distract the participant from a relentless focus on empathy. On each empathy item a person can score 2, 1, or 0, so the EQ has a maximum score of 80 (higher scores represent greater empathy). The EQ is able to pick up considerable individual, gender, and group differences, in both general-population and clinical samples.

#### 4.3 Twenty-Item toronto alexithymia scale (TAS-20)

The TAS-20 is the most commonly used measure of alexithymia, i.e. a deficit in the cognitive processing and regulation of emotion, resulting in difficulties in describing and identifying feelings [82]. The TAS-20 [83] is a validated self-report scale comprised of 20 items. People being assessed are asked to rank their own answers on a five-point Likert scale, with 5 indicating the highest degree of agreement and 1 the highest degree of disagreement with the affirmation expressed in each item. The TAS-20 has a three-factor structure: difficulty in identifying feelings (Factor 1, items 1, 3, 6, 7, 9, 13, 14), difficulty communicating feelings to other people (Factor 2, items 2, 4, 11, 12, 17), and externally oriented thinking (Factor 3, items 5, 8, 10, 15, 16, 18, 19, 20).

The total alexithymia score is the sum of responses to all 20 items. The TAS-20 cut-off scores are as follows: ≤51 no alexithymia, 52–60 borderline alexithymia, ≥61 alexithymia. This scale has shown good internal consistency and test-retest reliability, as well as convergent, discriminant and concurrent validity [84], and it is currently one of the most utilised instruments in alexithymia and emotion research.

#### 4.4 Multidimensional scale of perceived social support (MSPSS)

The MSPSS [85] is a validated self-report measure of subjectively assessed social support. This questionnaire provides assessment of three sources of social support: family, friends, and significant others. The MSPSS consists of 12 items, each scored on a seven-point Likert-type scale, easy to understand (requiring just a fourth-grade reading level) and suitable for young populations. The maximum score is 84; higher scores indicate greater perceived support. The MSPSS has been shown to be relatively free of social-desirability bias [86].

#### 4.5 Beck depression inventory, revised version (BDI-II)

The presence of symptoms related to depression was evaluated by means of the BDI-II, a 21-item multiple-choice self-rated scale [87]. The total score was obtained considering all items, rated from 0 to 3. A total scores of 0–13 indicates minimal depression, 14–19 mild depression, 20–28 moderate depression, and, finally, scores higher than 29 are indicative of severe depression.

#### 4.6 Eating attitude test (EAT)

The EAT [80] is the most broadly used self-report questionnaire in the field of eating disorders. The 40 items composing the EAT are presented in a six-point forced-choice Likert-type scale ranging from 1 (never) to 6 (always). Of these response options, three are scored with 1, 2 or 3, and the rest with 0. Total EAT score ranges from 0 to 120, and the clinical cut-off point is usually considered to be 30.

### 5. Statistical Analyses

Data were analysed using the Statistical Package for the Social Sciences (SPSS), version 18.0. Graphical and statistical exploration of the data by means of box plots, histograms, Q-Q plots and normality tests indicated a normal distribution of all measures; hence, parametric tests such as the unpaired t-test and Pearson’s correlation coefficients were used, as appropriate. All tests were two-tailed and were conducted at the 5% level of statistical significance.

## Results

Preliminary analyses revealed no differences between the participants of the AN-BP subtype and those of the AN-R subtype in any of the demographic, clinical, or experimental variables considered, except for age (AN-BP = 22.43, AN-R = 17.38; unpaired t-test, df = 28, *t* = 2.473, *p* = .020), education (AN-BP = 14.43, AN-R = 12.13; df = 28, *t = *2.384, *p* = .024) and duration of illness (AN-BP = 5.86, AN-R = 1.69; df = 28, *t* = 2.320, *p* = .028). These results are in line with those indicating that the AN patients of the restricting type tend to have a shorter duration of illness and to be younger (and consequently with less years of education) than those of the binge-purge subtype, and suggesting that the AN-R subtype represents a phase in the course of AN rather than a distinct subtype [7]–[8]. For this reason, and since no significant differences were found in any experimental variables considered, we collapsed the two subtypes of AN into a single group for further analyses.

### Descriptive and Clinical Data

Participants’ demographic and clinical data can be seen in Table 1. The two groups were comparable on age, years of education and height. Self-evidently, there were statistically significant differences for the weight and BMI variables. BMI ranged from 12.27 to 17.79 in the AN group and from 18.56 to 24.61 in the HC group. The AN group was significantly more depressed than the HC group, as measured by the BDI-II. According to the cut-off scores of the BDI-II, 33% of the patients with AN (10/30) had minimal or mild depression and 67% (20/30) had moderate or severe depression, compared with 94% (30/32) with minimal or mild depression and 6% (2/32) with moderate or severe depression in the HC group (data not shown in table). All of the participants in the HC group were well below the cut-off of the EAT, with a minimum of zero and a maximum of 21.

**Table 1:** Demographic and clinical characteristics.

	AN group Mean (SD)	HC group Mean (SD)	*t*-test results
	(n = 30)	(n = 32)	(df = 60)
Age, years	19.73 (6.06)	20.47 (2.72)	*t* = −.623, *p* = .536
Years of education	13.20 (2.85)	14.13 (1.76)	*t* = −1.551, *p* = .126
Height (cm)	162.11 (5.03)	164.66 (5.95)	*t* = −1.814, *p* = .075
Weight (kg)	39.54 (4.57)	54.78 (4.72)	*t* = −12.916, *p*<.001
Body Mass Index (Kg/m^2^)	15.06 (1.74)	20.21 (1.45)	*t* = −12.668, *p*<.001
Age of onset	15.77 (3.74)	–	–
Duration of illness	3.63 (5.27)	–	–
EAT (maximum score = 120, cut-off = 30)	–	7.59 (4.98)	–
BDI-II (maximum score = 63)	26.40 (13.39)	8.94 (6.66)	*t* = 6.563, *p*<.001

HC = Healthy controls, AN = Anorexia nervosa, SD = Standard deviation, df = Degrees of freedom, cm = centimeters, Kg = Kilograms, BMI = Body Mass Index, EAT = Eating Attitudes Test, BDI-II = Beck Depression Inventory-II.

### Social-cognition Tasks and Social-support Questionnaire

In table 2 are reported the participants’ scores to the ToM and emotional functioning tasks, and to the social-support questionnaire. For the RME, the number of correct responses for both the experimental (mental-state attribution) and the control (gender attribution) tasks were considered. Statistical analyses revealed the absence of significant differences on both the experimental and control tasks. The groups demonstrated significantly different results for both the EQ and TAS-20 tasks, with AN patients showing a lower degree of empathy and a higher degree of alexithymia. According to the cut-off scores of the TAS-20, 17% of the patients with AN (5/30) had borderline alexithymia and 47% (14/30) had high alexithymia, compared with 9% (3/32) with borderline alexithymia and no occurrence of high alexithymia in the HC group (data not shown in table). The group of patients with AN scored significantly higher than the HC group on all the three factors of the TAS-20. This result remains significant also after using the Bonferroni correction for multiple testing (.05/3). Finally, compared to the HC group, a lower level of perceived social support as assessed by the MSPSS was found in the AN group.

**Table 2:** Participants’ scores to the tasks assessing ToM and emotional functioning, and to the social-support questionnaire.

	AN group Mean (SD)	HC group Mean (SD)	*p* value
	(n = 30)	(n = 32)	(df = 60)
*ToM and emotional functioning tasks*			
RME experimental (maximum score = 36)	25.60 (3.93)	26.97 (3.33)	*t* = −1.484, *p* = .143
RME control (maximum score = 36)	34.20 (1.79)	34.69 (1.45)	*t* = −1.183, *p* = .241
EQ (maximum score = 80)	44.17 (11.47)	50.72 (8.35)	*t* = −2.584, *p* = .012
TAS-20 Global score (maximum score = 100)	56.70 (12.94)	40.47 (7.77)	*t* = 6.032, *p*<.001
TAS-20, Factor 1 (max. score = 35)	21.30 (6.61)	14.00 (3.19)	*t* = 5.954, *p*<.001
TAS-20, Factor 2 (max. score = 25)	16.80 (4.57)	12.06 (3.35)	*t* = 4.678, *p*<.001
TAS-20, Factor 3 (max. score = 40)	18.60 (5.63)	14.41 (3.56)	*t* = 3.528, *p* = .001
*Social-support questionnaire*			
MSPSS (maximum score = 84)	68.27 (12.48)	75.03 (5.63)	*t* = −2.780, *p* = .007

HC = Healthy controls, AN = Anorexia nervosa, ToM = Theory of Mind, SD = Standard deviation, df = Degrees of freedom, RME = Reading the Mind in the Eyes, EQ = Empathy Quotient, TAS-20 = Twenty-item Toronto Alexithymia Scale, EAT = Eating Attitudes Test, MSPSS = Multidimensional Scale of Perceived Social Support.

### Correlation Analyses

The correlation analyses for the AN group are shown in Table 3. Given the exploratory nature of these analyses, there is no correction for multiple comparisons. No significant correlations were found between BMI, age of onset, or duration of illness and any of the clinical or experimental tasks. Equally, no correlations were found between the RME task and any of the other measures. A positive correlation was found between TAS-20 and BDI-II, whereas negative correlations was found between EQ and TAS-20 and between BDI-II and MSPSS. Interestingly, for what concerns the MSPSS, a positive correlation was found with EQ and a negative correlation with TAS-20, even when controlling for BMI, BDI-II, age of onset, and duration of illness (partial correlations, *r* = .593 *p* = .001 and *r* = −463 *p* = .017, respectively).

**Table 3:** Pearson correlations in the AN group. Significant correlations are shown in bold.

	BMI	Onset	Duration	BDI-II	RME	TAS-20	EQ	MSPSS
BMI	–							
Onset	*r* = .103*p* = .589	–						
Duration	*r* = .013*p* = .947	*r* = −.136*p* = .474	–					
BDI-II	*r* = .172*p* = .363	*r* = −.277*p* = .138	*r* = −.063*p* = .740	*–*				
RME	*r* = .020*p* = .917	*r* = −.178*p* = .347	*r* = .301*p* = .106	*r* = .102*p* = .591	*–*			
TAS-20	*r* = .173*p* = .361	*r* = .146*p* = .441	*r* = −.249*p* = .185	***r*** ** = .523** ***p*** ** = .003**	*r* = −.059*p* = .755	*–*		
EQ	*r* = .038*p* = .840	*r* = −.139*p* = .464	*r* = .302*p* = .104	*r* = −.038*p* = .844	*r* = .172*p* = .365	***r*** ** = **−**.471** ***p*** ** = .009**	*–*	
MSPSS	*r* = −.270*p* = .149	*r* = .084*p* = .658	*r* = .200*p* = .290	***r*** ** = **−**.436** ***p*** ** = .016**	*r* = .135*p* = .478	***r*** ** = **−**.587** ***p*** ** = .001**	***r*** ** = .539** ***p*** ** = .002**	*–*

BMI = Body Mass Index, BDI-II = Beck Depression Inventory-II, RME = Reading the Mind in the Eyes, TAS-20 = Twenty-item Toronto Alexithymia Scale, EQ = Empathy Quotient, MSPSS = Multidimensional Scale of Perceived Social Support.

## Discussion

The main aim of the present study was to investigate social cognition in patients with AN. The pattern of results followed the experimental hypothesis: more precisely, the performance of patients with AN is significantly worse than that of healthy controls on tasks assessing the emotional functioning (i.e. EQ and TAS-20), whereas patients’ performance is comparable to that of healthy controls on the ToM task (i.e. RME). Interestingly, correlation analyses showed no relationship between scores on any of the social-cognition tasks and either age of onset or duration of illness.

Our results are in line with previous studies showing emotional functioning deficits in people with AN [44]–[47] and unimpaired performance in the ToM domain [28], [31]. On the contrary, we found divergent results from those of Russell et al. [33]: these authors used the RME to test the ToM ability in a sample of 22 English female participants with AN and found that they performed more poorly than the HC group. Although slight cultural differences between their sample and the one investigated in the present study (composed of Italian females with AN) may have influenced responses to the RME at least to some degree, we believe that the difference between the results of these two studies is mainly due to methodological issues. First of all, in the study by Russell et al. [33] there was a statistically significant difference between the AN and HC groups on both mean age and years of education. It is now well known that mean age is a particularly important variable when assessing ToM ability with the RME, as different studies have recently found age-related effects on performance in this task [88]–[90] and a neuroimaging study has demonstrated age-related differences in the neural activation associated with the RME in adolescents [91]. Education is another relevant variable, as it seems reasonable to expect that average length of educational experience might potentially impact understanding of the target word and the three foil words. Until now, it has never been clearly shown that scores obtained on this test do not strongly depend on the individual’s general intelligence [92]; it has even been suggested that intelligence quotient may predict performance on the RME in AN patients [34]. For these reasons, we suggest that future research should pay particular attention to the participants’ demographic variables, avoiding comparisons between the performance of samples that are not well matched for age or education. Furthermore, in the present study we assigned to the HC group only participants with a BMI between 18.50 and 24.99 (i.e. within the normal range of weight according to the World Health Organization) and a score below the cut-off of 30 on the EAT (i.e. without the risk of having an eating disorder). In the study by Russell et al. [33] the HC group had a mean BMI of 26.2, and no test to check for potential eating disorders was administered to this group.

A further variable that may contribute to explain discrepancies between our results on the ToM task and those of previous studies is the duration of illness. While in our group of participants with AN the mean duration of illness is well below four years, in the study by Russell et al. [33] this variable exceeds nine years, as well as in the studies by Harrison et al. [35]–[36] in which a statistical difference to the RME was found. As suggested by Harrison et al. [35], it is possible that their findings may be secondary to severe weight loss and the starvation effects associated with AN and that it would be a good idea to explore this question using a sample with a shorter illness duration. We have followed this suggestion. To the best of our knowledge, no previous studies have investigated the ToM ability in an AN group with a shorter duration of illness and thus the present findings contribute to extend our knowledge on the relationship between this latter dimension and the level of ToM ability in patients with AN. We suggest that this relationship should be further investigated in longitudinal studies that could properly test the hypothesis proposed by Zucker et al. [52] according to which individuals with AN are characterised by a chronic avoidance of salient social cues (such as facial ones) that leads to increased dysfunction over time.

We interpret our results also in light of the evidence of the crucial role played by the amygdala in the neurobiology of AN. In fact, numerous structural and functional neuroimaging studies [68]–[77] point to the role of this cerebral region in emotional processing and to its alterations in people with AN, supporting our hypothesis of impaired performance among these people in the tasks we used to assess emotional functioning. While the role of the amygdala in determining the capacity to share emotional experiences and to recognize emotions in oneself and others is well known and acknowledged [55]–[56], [66]–[67], the role of this brain structure in ToM is very controversial and highly debated. Indeed, although Baron-Cohen et al. [93] proposed the ‘amygdala theory’ to explain autism (a neurodevelopmental disorder characterised by ToM impairments), this theory has been subsequently revisited by Dziobek et al. [63], who show that in autism the amygdala is not crucially involved in social functioning, and by Paul et al. [64] who by assessing two rare individuals with developmental bilateral amygdalar lesions demonstrated that the amygdala is not essential for the aspects of social behavior that are diagnostically characteristic of autism. Furthermore, investigating by means of a factorial design the interaction between the emotional and perspective-tacking (ToM) factors, Ruby and Decety [65] come to the conclusion that the amygdala is not necessary for ToM expression. Anyway, it is worth noting that with only one exception [94] none of the functional imaging studies that have to date investigated the ToM brain areas involved in solving the RME task report amygdala activations [91], [95]–[98] and that a lesion study examining two patients with bilateral amygdalar damage reported impaired performance to the RME in only one patient [99]. Overall, these findings seem to support the hypothesis originally proposed by Gallagher and Frith [62] according to which the amygdala is not involved in mentalising *per se*, as well as our hypothesis of a preserved ToM ability in patients with AN on the RME task.

Our results concerning perceived social support deserve attention. They are in accordance with previous findings reporting lower levels of perceived social support in patients with AN than in an HC group [100] and showing no correlation between social support and duration of illness [53]. Furthermore, to the best of our knowledge, the present study is the first to demonstrate a correlation between social support as perceived by AN patients and emotional functioning. Although the nature of the present study does not permit inference of cause and effect, this latter result is particularly relevant, as it seems to suggest a potential role of social support in the treatment and recovery of AN, and it supports previous indications according to which interventions designed to encourage patients to broaden their social networks may be a useful addition to standard approaches based on a good therapeutic relationship [53], [101]. In particular, interventions based on helping the patient to reflect on the impact her behaviors have on the feelings of her carers and, contextually, based on developing more effective methods for eliciting social support might be recommended.

The present study has several limitations, however. First, compared with other studies in this area, we enrolled a greater number of patients with AN, but nonetheless, our study is still limited by a relatively small sample size. Second, although there is a strong amount of structural and functional evidence supporting the crucial role of the amygdala in the neurobiology of AN, we conducted no direct measurement of activity in this brain structure in the patients of our sample. Third, the experimental tasks we used elicit mainly reflective processes of social cognition. Further studies should integrate this kind of task with others able to elicit more automatic processes as well: as face regions outside from the eyes can convey a number of socially relevant information, of particular interest are the tasks concerning the evaluation of faces expressing basic and social emotions [102]–[106]. Despite these limitations, we believe the findings presented here may be useful to help us better understand the social-cognitive profile of patients with AN and to contribute to the development of effective interventions based on the ways in which patients with AN actually perceive their social environment.

## References

[1] American Psychiatric Association (2000) Diagnostic and statistical manual of mental disorders, 4th ed. Washington, DC: APA.

[2] Davis C, Blackmore E, Katzman DK, Fox J (2005) Female adolescents with anorexia nervosa and their parents: A case-control study of exercise attitudes and behaviours. Psychol Med 35: 377–386. 15841873 10.1017/s0033291704003447

[3] Keating C (2010) Theoretical perspective on anorexia nervosa: The conflict of reward. Neurosci Biobehav Rev 34: 73–79. 19619579 10.1016/j.neubiorev.2009.07.004

[4] Connan F, Campbell I, Katzman M, Lightman S, Treasure J (2003) A neurodevelopmental model for anorexia nervosa. Physiol Behav 79: 13–24. 12818706 10.1016/s0031-9384(03)00101-x

[5] Morris J, Twaddle S (2007) Anorexia nervosa. BMJ 334: 894–898. 17463461 10.1136/bmj.39171.616840.BE PMC1857759

[6] Hoek HW (2006) Incidence, prevalence and mortality of anorexia nervosa and other eating disorders. Curr Opin Psychiatry 19: 389–394. 16721169 10.1097/01.yco.0000228759.95237.78

[7] Eddy KT, Dorer DJ, Franko DL, Tahilani K, Thompson-Brenner H, et al (2008) Diagnostic crossover in anorexia nervosa and bulimia nervosa: Implication for DSM-V. Am J Psychiatry 165: 245–250. 18198267 10.1176/appi.ajp.2007.07060951 PMC3684068

[8] Eddy KT, Keel PK, Dorer DJ, Delinsky SS, Franko DL, et al (2002) Longitudinal comparison of anorexia nervosa subtypes. Int J Eat Disord 31: 191–201. 11920980 10.1002/eat.10016

[9] Bara BG, Ciaramidaro A, Walter H, Adenzato M (2011) Intentional minds: A philosophical analysis of intention tested through fMRI experiments involving people with schizophrenia, people with autism, and healthy individuals. Front Hum Neurosci 5: 7 doi: 10.3389/fnhum.2011.00007. 21344005 10.3389/fnhum.2011.00007 PMC3034216

[10] Cheng YW, Chou KH, Fan YT, Lin CP (2011) ANS: Aberrant Neurodevelopment of the Social cognition network in adolescents with autism spectrum disorders. PLoS One 6(4): e18905. 21541322 10.1371/journal.pone.0018905 PMC3082537

[11] Di Martino A, Ross K, Uddin LQ, Sklar AB, Castellanos FX, et al (2009) Functional brain correlates of social and nonsocial processes in autism spectrum disorders: An activation likelihood estimation meta-analysis. Biol Psychiatry 65: 63–74. 18996505 10.1016/j.biopsych.2008.09.022 PMC2993772

[12] Sugranyes G, Kyriakopoulos M, Corrigall R, Taylor E, Frangou S (2011) Autism spectrum disorders and schizophrenia: Meta-analysis of the neural correlates of social cognition. PLoS One 6(10): e25322. 21998649 10.1371/journal.pone.0025322 PMC3187762

[13] Walter H, Ciaramidaro A, Adenzato M, Vasic N, Ardito RB, et al (2009) Dysfunction of the social brain in schizophrenia is modulated by intention type: An fMRI study. Soc Cogn Affect Neurosci 4: 166–176. 19287044 10.1093/scan/nsn047 PMC2686225

[14] Adenzato M, Cavallo M, Enrici I (2010) Theory of Mind ability in the behavioural variant of frontotemporal dementia: An analysis of the neural, cognitive, and social levels. Neuropsychologia 48: 2–12. 19666039 10.1016/j.neuropsychologia.2009.08.001

[15] Calabria M, Cotelli M, Adenzato M, Zanetti O, Miniussi C (2009) Empathy and emotion recognition in semantic dementia: A case report. Brain Cogn 70: 247–252. 19339096 10.1016/j.bandc.2009.02.009

[16] Cavallo M, Adenzato M, MacPherson SE, Karwig G, Enrici I, et al (2011) Evidence of social understanding impairment in patients with Amyotrophic Lateral Sclerosis. PLoS One 6(10): e25948. 21998727 10.1371/journal.pone.0025948 PMC3187828

[17] Poletti M, Enrici I, Adenzato M (2012) Cognitive and affective Theory of Mind in neurodegenerative diseases: Neuropsychological, neuroanatomical and neurochemical levels. Neurosci Biobehav Rev doi: 10.1016/j.neubiorev.2012.07.004. 10.1016/j.neubiorev.2012.07.004 22819986

[18] Poletti M, Enrici I, Bonuccelli U, Adenzato M (2011) Theory of Mind in Parkinson’s disease. Behav Brain Res 219: 342–350. 21238496 10.1016/j.bbr.2011.01.010

[19] Adolphs R (2001) The neurobiology of social cognition. Curr Opin Neurobiol 11: 231–239. 11301245 10.1016/s0959-4388(00)00202-6

[20] Adolphs R (2003) Investigating the cognitive neuroscience of social behaviour. Neuropsychologia 41: 119–126. 12459210 10.1016/s0028-3932(02)00142-2

[21] Frith CD, Frith U (2007) Social cognition in humans. Curr Biol 17: R724–R732. 17714666 10.1016/j.cub.2007.05.068

[22] Leslie AM (1987) Pretense and representation: The origins of ‘Theory of Mind’. Psychol Rev 94: 412–426.

[23] Premack D, Woodruff G (1978) Does the chimpanzee have a Theory of Mind? Behav Brain Sci 1: 515–526.

[24] Decety J, Jackson PL (2004) The functional architecture of human empathy. Behav Cogn Neurosci Rev 3: 71–100. 15537986 10.1177/1534582304267187

[25] Lieberman MD (2007) Social cognitive neuroscience: A review of core processes. Annu Rev Psychol 58: 259–289. 17002553 10.1146/annurev.psych.58.110405.085654

[26] Adenzato M, Garbarini F (2006) The As If in cognitive science, neuroscience and anthropology: A journey among robots, blacksmiths, and neurons. Theory Psychol 16: 747–759.

[27] Singer T (2006) The neuronal basis and ontogeny of empathy and mind reading: Review of literature and implications for future research. Neurosci Biobehav Rev 30: 855–863. 16904182 10.1016/j.neubiorev.2006.06.011

[28] Tchanturia K, Happé F, Godley J, Treasure J, Bara-Carril N, et al (2004) ‘Theory of Mind’ in anorexia nervosa. Eur Eat Disord Rev 12: 361–366.

[29] Happé F (1994) An advanced test of theory of mind: Understanding of story characters’ thoughts and feelings by able autistic, mentally handicapped and normal children and adults. J Autism Dev Disord 24: 129–154. 8040158 10.1007/BF02172093

[30] Happé F, Brownell H, Winner E (1999) Acquired ‘theory of mind’ impairments following stroke. Cognition 70: 211–240. 10384736 10.1016/s0010-0277(99)00005-0

[31] Hambrook D, Tchanturia K (2008) A pilot study exploring Machiavellianism in anorexia nervosa. Eat Weight Disord 13: 137–141. 19011371 10.1007/BF03327614

[32] Christie R, Geis FL (1970) Studies in Machiavellianism. New York: Academic Press.

[33] Russell TA, Schmidt U, Doherty L, Young V, Tchanturia K (2009) Aspects of social cognition in anorexia nervosa: Affective and cognitive theory of mind. Psychiatry Res 168: 181–185. 19467562 10.1016/j.psychres.2008.10.028

[34] Oldershaw A, Hambrook D, Tchanturia K, Treasure J, Schmidt U (2010) Emotional Theory of Mind and emotional awareness in recovered anorexia nervosa patients. Psychosom Med 72: 73–79. 19995886 10.1097/PSY.0b013e3181c6c7ca

[35] Harrison A, Sullivan S, Tchanturia K, Treasure J (2009) Emotion recognition and regulation in anorexia nervosa. Clin Psychol Psychother 16: 348–356. 19517577 10.1002/cpp.628

[36] Harrison A, Tchanturia K, Treasure J (2010) Attentional bias, emotion recognition, and emotion regulation in anorexia: State or trait? Biol Psychiatry 68: 755–761. 20591417 10.1016/j.biopsych.2010.04.037

[37] Harrison A, Sullivan S, Tchanturia K, Treasure J (2010) Emotional functioning in eating disorders: Attentional bias, emotion recognition and emotion regulation. Psychol Med 40: 1887–1897. 20102669 10.1017/S0033291710000036

[38] Hambrook D, Tchanturia K, Schmidt U, Russell T, Treasure J (2008) Empathy, systemizing, and autistic traits in anorexia nervosa: A pilot study. Br J Clin Psychol 47: 335–339. 18208640 10.1348/014466507X272475

[39] Baron-Cohen S, Wheelwright S, Skinner R, Martin J, Clubley E (2001) The autism spectrum quotient (AQ): Evidence from Asperger syndrome/high functioning autism, males and females, scientists and mathematicians. J Autism Dev Disord 31: 5–17. 11439754 10.1023/a:1005653411471

[40] Baron-Cohen S, Richler J, Bisarya D, Gurunathan N, Wheelwright S (2003) The systemizing quotient (SQ): An investigation of adults with Asperger syndrome or high functioning autism and normal sex differences. Philos Trans R Soc Lond B Biol Sci 358: 361–374. 12639333 10.1098/rstb.2002.1206 PMC1693117

[41] Baron-Cohen S, Wheelwright S (2004) The empathy quotient: An investigation of adults with Asperger syndrome or high functioning autism, and normal sex differences. J Autism Dev Disord 34: 163–175. 15162935 10.1023/b:jadd.0000022607.19833.00

[42] Guttman HA, Laporte L (2000) Empathy in families of women with borderline personality disorder, anorexia nervosa, and a control group. Fam Process 39: 345–358. 11008652 10.1111/j.1545-5300.2000.39306.x

[43] Davis MH (1983) Measuring individual differences in empathy: Evidence for a multidimensional approach. J Pers Soc Psychol 44: 113–126.

[44] Gillberg IC, Rastam M, Gillberg C (1995) Anorexia nervosa six years after onset: Part I. Personality disorders. Compr Psychiatry 36: 61–69. 7705090 10.1016/0010-440x(95)90100-a

[45] Bourke MP, Taylor GJ, Parker JDA, Bagby RM (1992) Alexithymia in women with anorexia nervosa: A preliminary investigation. Br J Psychiatry 161: 240–243. 1521107 10.1192/bjp.161.2.240

[46] Schmidt U, Jiwany A, Treasure J (1993) A controlled study of alexithymia in eating disorders. Compr Psychiatry 34: 54–58. 8425393 10.1016/0010-440x(93)90036-4

[47] Speranza M, Loas G, Wallier J, Corcos M (2007) Predictive value of alexithymia in patients with eating disorders: A 3-year prospective study. J Psychosom Res 63: 365–370. 17905043 10.1016/j.jpsychores.2007.03.008

[48] Kucharska-Pietura K, Nikolaou V, Masiak M (2004) The recognition of emotion in the faces and voice of anorexia nervosa. Int J Eat Disord 35: 42–47. 14705156 10.1002/eat.10219

[49] Pollatos O, Herbert BM, Schandry R, Gramann K (2008) Impaired central processing of emotional faces in anorexia nervosa. Psychosom Med 70: 701–708. 18606730 10.1097/PSY.0b013e31817e41e6

[50] Zonnevijlle-Bendek MJS, van Goozen SHM, Cohen-Kettenis PT, van Elburg A, van Engeland H (2002) Do adolescent anorexia nervosa patients have deficits in emotional functioning? Eur Child Adolesc Psychiatry 11: 38–42. 11942427 10.1007/s007870200006

[51] Rozenstein MH, Latzer Y, Stein D, Eviatar Z (2011) Perception of emotion and bilateral advantage in women with eating disorders, their healthy sisters, and nonrelated healthy controls. J Affect Disord 134: 386–395. 21757238 10.1016/j.jad.2011.06.009

[52] Zucker NL, Losh M, Bilik C, LaBar KS, Piven J, et al (2007) Anorexia nervosa and autistic spectrum disorders: A guided investigation of social cognitive endophenotypes. Psychol Bull 113: 976–1006. 10.1037/0033-2909.133.6.976 17967091

[53] Tiller JM, Sloane G, Schmidt U, Troop N, Power M, et al (1997) Social support in patients with anorexia nervosa and bulimia nervosa. Int J Eat Disord 21: 31–38. 8986515 10.1002/(sici)1098-108x(199701)21:1<31::aid-eat4>3.0.co;2-4

[54] Decety J (2010) The neurodevelopment of empathy in human. Dev Sci 32: 257–267. 10.1159/000317771 PMC3021497 20805682

[55] Lee KH, Siegle GJ (2012) Common and distinct brain networks underlying explicit emotional evaluation: A meta-analytic study. Soc Cogn Affect Neurosci 7: 521–534. 19270039 10.1093/scan/nsp001 PMC3375881

[56] Vollm BA, Taylor AN, Richardson P, Corcoran R, Stirling J, et al (2006) Neuronal correlates of theory of mind and empathy: A functional magnetic resonance imaging study in a nonverbal task. NeuroImage 29: 90–98. 16122944 10.1016/j.neuroimage.2005.07.022

[57] Ciaramidaro A, Adenzato M, Enrici I, Erk S, Pia L, Bara BG, Walter H (2007) The intentional network: How the brain reads varieties of intentions. Neuropsychologia 45: 3105–3113. 17669444 10.1016/j.neuropsychologia.2007.05.011

[58] Enrici I, Adenzato M, Cappa S, Bara BG, Tettamanti M (2011) Intention processing in communication: A common brain network for language and gestures. J Cogn Neurosci 23: 2415–2431. 20954937 10.1162/jocn.2010.21594

[59] Walter H, Adenzato M, Ciaramidaro A, Enrici I, Pia L, et al (2004) Understanding intentions in social interactions: The role of the anterior parancingulate cortex. J Cogn Neurosci 16: 1854–1863. 15701234 10.1162/0898929042947838

[60] Schnell K, Bluschke S, Konradt B, Walter H (2011) Functional relations of empathy and mentalizing: An fMRI study on the neural basis of cognitive empathy. NeuroImage 54: 1743–1754. 20728556 10.1016/j.neuroimage.2010.08.024

[61] Van Overwalle F (2009) Social cognition and the brain: A meta-analysis. Hum Brain Mapp 30: 829–858. 18381770 10.1002/hbm.20547 PMC6870808

[62] Gallagher HL, Frith CD (2003) Functional imaging of 'theory of mind'. Trends Cogn Sci 7: 77–83. 12584026 10.1016/s1364-6613(02)00025-6

[63] Dziobek I, Fleck S, Rogers K, Wolf OT, Convit A (2006) The 'amygdala theory of autism' revisited: Linking structure to behavior. Neuropsychologia 44: 1891–1899. 16566949 10.1016/j.neuropsychologia.2006.02.005

[64] Paul LK, Corsello C, Tranel D, Adolphs R (2010) Does bilateral damage to the human amygdala produce autistic symptoms? J Neurodevelop Disord 2: 165–173. 10.1007/s11689-010-9056-1 PMC2914867 20700516

[65] Ruby P, Decety J (2004) How would you feel versus how do you think she would feel? A neuroimaging study of perspective-taking with social emotions. J Cogn Neurosci 16: 988–999. 15298786 10.1162/0898929041502661

[66] Costafreda SG, Brammer MJ, David AS, Fu CH (2008) Predictors of amygdala activation during the processing of emotional stimuli: A meta-analysis of 385 PET and fMRI studies. Brain Res Rev 58: 57–70. 18076995 10.1016/j.brainresrev.2007.10.012

[67] Phan KL, Wager T, Taylor SF, Liberzon I (2002) Functional neuroanatomy of emotion: A meta-analysis of emotion activation studies in PET and fMRI. NeuroImage 16: 331–348. 12030820 10.1006/nimg.2002.1087

[68] Ellison Z, Foong J, Howard R, Bullmore E, Williams S, et al (1998) Functional anatomy of calorie fear in anorexia nervosa. Lancet 352: 1192. 10.1016/S0140-6736(05)60529-6 9777839

[69] Friederich HC, Walther S, Bendszus M, Biller A, Thomann P, et al (2012) Grey matter abnormalities within cortico-limbic-striatal circuits in acute and weight-restored anorexia nervosa patients. NeuroImage 59: 1106–1113. 21967727 10.1016/j.neuroimage.2011.09.042

[70] Giordano GD, Renzetti P, Parodi RC, Foppiani L, Zandrino F, et al (2001) Volume measurement with magnetic resonance imaging of hippocampus-amygdala formation in patients with anorexia nervosa. J Endocrinol Invest 24: 510–514. 11508785 10.1007/BF03343884

[71] Joos AAB, Saum B, van Elst LT, Perlov E, Glauche V, et al (2011) Amygdala hyper-reactivity in restrictive anorexia nervosa. Psychiatry Res: Neuroimaging 191: 189–195. 21316204 10.1016/j.pscychresns.2010.11.008

[72] Miyake Y, Okamoto Y, Onoda K, Kurosaki M, Shirao N, et al (2010) Brain activation during the perception of distorted body images in eating disorders. Psychiatry Res: Neuroimaging 181: 183–192. 20153150 10.1016/j.pscychresns.2009.09.001

[73] Miyake Y, Okamoto Y, Onoda K, Shirao N, Okamoto Y, et al (2010) Neural processing of negative word stimuli concerning body image in patients with eating disorders: An fMRI study. NeuroImage 50: 1333–1339. 20045473 10.1016/j.neuroimage.2009.12.095

[74] Seeger G, Braus DF, Ruf M, Goldberger U, Schmidt MH (2002) Body image distortion reveals amygdala activation in patients with anorexia nervosa: A functional magnetic resonance imaging study. Neurosci Lett 326: 25–28. 12052530 10.1016/s0304-3940(02)00312-9

[75] Takano A, Shiga T, Kitagawa N, Koyama T, Katoh C, et al (2001) Abnormal neuronal network in anorexia nervosa studied with I-123-IMP SPECT. Psychiatry Res: Neuroimaging 107: 45–50. 11472863 10.1016/s0925-4927(01)00093-2

[76] Vocks S, Busch M, Gronemeyer D, Schulte D, Herpertz S, et al (2010) Neural correlates of viewing photographs of one's own body and another woman's body in anorexia and bulimia nervosa: An fMRI study. J Psychiatry Neurosci 35: 163–176. 20420767 10.1503/jpn.090048 PMC2861133

[77] Vocks S, Herpertz S, Rosenberger C, Senf W, Gizewski ER (2010) Effects of gustatory stimulation on brain activity during hunger and satiety in females with restricting-type anorexia nervosa: An fMRI study. J Psychiatr Res 45: 395–403. 20709330 10.1016/j.jpsychires.2010.07.012

[78] Spitzer RL (1994) Structured Clinical Interview for DSM-IV. Washington, DC: APA.

[79] World Health Organization (1995) Physical status: The use and interpretation of anthropometry. Report of a WHO Expert Committee. WHO Tech Rep Ser 854: 1–452. 8594834

[80] Garner DM, Garfinkel PE (1979) The Eating Attitudes Test: An index of the symptoms of anorexia nervosa. Psychol Med 9: 273–279. 472072 10.1017/s0033291700030762

[81] Baron-Cohen S, Wheelwright S, Hill J, Raste Y, Plumb I (2001) The "Reading the Mind in the Eyes" test revised version: A study with normal adults, and adults with Asperger syndrome or high-functioning autism. J Child Psychol Psychiatry 42: 241–251. 11280420

[82] Taylor GJ, Bagby RM, Parker JDA (1997) Disorders of affect regulation: Alexithymia in medical and psychiatric illness. Cambridge, UK: Cambridge University Press.

[83] Bagby RM, Parker JDA, Taylor GJ (1994) The Twenty-item Toronto Alexithymia Scale 1: Item selection and cross-validation of the factor structure. J Psychosom Res 38: 23–32. 8126686 10.1016/0022-3999(94)90005-1

[84] Bagby RM, Taylor GJ, Parker JDA (1994) The Twenty-item Toronto Alexithymia Scale 2: Convergent, discriminant, and concurrent validity. J Psychosom Res 38: 33–40. 8126688 10.1016/0022-3999(94)90006-x

[85] Zimet GD, Dahlem NW, Zimet SG, Farley GK (1988) The multidimensional scale of perceived social support. J Pers Assess 52: 30–41. 10.1080/00223891.1990.9674095 2280326

[86] Dahlem NW, Zimet GD, Walker RR (1991) The multidimensional scale of perceived social support: A confirmation study. J Clin Psychol 47: 756–761. 1757578 10.1002/1097-4679(199111)47:6<756::aid-jclp2270470605>3.0.co;2-l

[87] Beck AT, Steer RA, Ball R, Ranieri W (1996) Comparison of Beck Depression Inventories -IA and -II in psychiatric outpatients. J Pers Assess 67: 588–597. 8991972 10.1207/s15327752jpa6703_13

[88] Bailey PE, Henry JD, Von Hippel W (2008) Empathy and social functioning in late adulthood. Aging Ment Health 12: 499–503. 18791898 10.1080/13607860802224243

[89] Duval C, Piolino P, Bejanin A, Eustache F, Desgranges B (2011) Age effect on different components of theory of mind. Conscious Cogn 20: 627–642. 21111637 10.1016/j.concog.2010.10.025

[90] Slessor G, Phillips LH, Bull R (2007) Exploring the specificity of age-related differences in theory of mind tasks. Psychol Aging 22: 639–643. 17874961 10.1037/0882-7974.22.3.639

[91] Moor BG, de Macks ZAO, Guroglu B, Rombouts SARB, Van der Molen MW, et al (2012) Neurodevelopmental changes of reading the mind in the eyes. Soc Cogn Affect Neurosci 7: 44–52. 21515640 10.1093/scan/nsr020 PMC3252628

[92] Hallerback MU, Lugnegard T, Hjarthag F, Gillberg C (2009) The Reading the Mind in the Eyes test: Test-retest reliability of a Swedish version. Cogn Neuropsychiatry 14: 127–143. 19370436 10.1080/13546800902901518

[93] Baron-Cohen S, Ring HA, Bullmore ET, Wheelwright S, Ashwin C, et al (2000) The amygdala theory of autism. Neurosci Biobehav Rev 24: 355–364. 10781695 10.1016/s0149-7634(00)00011-7

[94] Baron-Cohen S, Ring H, Wheelwright S, Bullmore ET, Brammer MJ, et al (1999) Social intelligence in the normal and autistic brain: An fMRI study. Eur J Neurosci 11: 1891–1898. 10336657 10.1046/j.1460-9568.1999.00621.x

[95] Adams RB, Rule NO, Franklin RG, Wang E, Stevenson MT, et al (2009) Cross-cultural reading the mind in the eyes: An fMRI investigation. J Cogn Neurosci 22: 98–108. 10.1162/jocn.2009.21187 19199419

[96] Baron-Cohen S, Ring H, Chitnis X, Wheelwright S, Gregory L, et al (2006) fMRI of parents of children with Asperger syndrome: A pilot study. Brain Cogn 61: 122–130. 16460858 10.1016/j.bandc.2005.12.011

[97] Platek SM, Keenan JP, Gallup GG Jr, Mohamed FB (2004) Where am I? The neurological correlates of self and other. Cogn Brain Res 19: 114–122. 10.1016/j.cogbrainres.2003.11.014 15019708

[98] Russell TA, Rubia K, Bullmore ET, Soni W, Suckling J, et al (2000) Exploring the social brain in schizophrenia: Left prefrontal underactivation during mental state attribution. Am J Psychiatry 157: 2040–2042. 11097974 10.1176/appi.ajp.157.12.2040

[99] Stone VE, Baron-Cohen S, Calder A, Keane J, Young A (2003) Acquired theory of mind impairments in individuals with bilateral amygdala lesions. Neuropsychologia 41: 209–220. 12459219 10.1016/s0028-3932(02)00151-3

[100] Kim YR, Lim SJ, Treasure J (2011) Different patterns of emotional eating and visuospatial deficits whereas shared risk factors related with social support between anorexia nervosa and bulimia nervosa. Psychiatry Investig 8: 9–14. 10.4306/pi.2011.8.1.9 PMC3079192 21519531

[101] Ardito RB, Rabellino D (2011) Therapeutic alliance and outcome of psychotherapy: Historical excursus, measurements, and prospects for research. Front Psychol 2: 270 doi: 10.3389/fpsyg.2011.00270. 22028698 10.3389/fpsyg.2011.00270 PMC3198542

[102] Adenzato M, Ardito RB, Izard E (2006) Impact of maternal directiveness and overprotectiveness on the personality development of a sample of individuals with acquired blindness. Soc Behav Pers 34: 17–26.

[103] Stewart LH, Ajina S, Getov S, Bahrami B, Todorov A, et al. (in press) Unconscious evaluation of faces on social dimensions. J Exp Psychol Gen doi: 10.1037/a0027950. 10.1037/a0027950 PMC3496403 22468670

[104] Tamietto M, Adenzato M, Geminiani G, de Gelder B (2007) Fast recognition of social emotions takes the whole brain: Interhemispheric cooperation in the absence of cerebral asymmetry. Neuropsychologia 45: 836–843. 16996092 10.1016/j.neuropsychologia.2006.08.012

[105] Tamietto M, Latini Corazzini L, de Gelder B, Geminiani G (2006) Functional asymmetry and interhemispheric cooperation in the perception of emotions from facial expressions. Exp Brain Res 171: 389–404. 16374630 10.1007/s00221-005-0279-4

[106] Young A, Perrett D, Calder A, Sprengelmeyer R, Ekman P (2002) Facial Expressions of Emotion - Stimuli and Tests (FEEST). Bury St Edmunds, UK: Thames Valley Test Company.

